# Strengthening capacity for cancer research in conflict settings: key informant insights from the Middle East

**DOI:** 10.3332/ecancer.2020.1153

**Published:** 2020-12-10

**Authors:** Zahi Abdul-Sater, Marilyne Menassa, Nassim El Achi, Rima A. Abdul-Khalek, Ghassan Abu-Sittah, Deborah Mukherji

**Affiliations:** 1 Global Health Institute, American University of Beirut, Beirut, Lebanon; 2 Naef K Basile Cancer Institute, Faculty of Medicine, American University of Beirut, Beirut, Lebanon

**Keywords:** cancer research, capacity strengthening, MENA region

## Abstract

**Background:**

Management of cancer in the Middle East and North Africa (MENA) region is accompanied by multiple challenges including heterogeneous access to early detection and treatment options and limited implementation of national cancer control plans. Furthermore, protracted armed conflicts across the region have had dramatic effects, including disruption of healthcare systems and the migration of healthcare professionals. Strengthening capacity for cancer research has been identified as a key intervention to correct data poverty, inform policy, manage limited resources and improve health outcomes.

**Objective:**

The main objective of this study is to gain insights into the landscape, barriers and enablers of cancer training, research and care in the MENA region.

**Method:**

We utilised purposive sampling to interview 16 key informants from a diverse academic, medical and research background originating from countries affected by conflicts, such as Lebanon, and from active conflict zones including Iraq and Syria.

**Results:**

The themes that emerged from the interviews focused on the barriers to cancer care, barriers to cancer research and training, strengths and importance of cancer research and training recommendations. The detrimental effect of conflict on cancer provision and research was a cross-cutting sub-theme disrupting cancer care provision and research due to unsafe environments, fragmented facilities, absence of drugs and migration of personnel. When asked about perceived optimal training format for cancer research, most informants recommended a post-graduate, face-to-face training focusing on cancer research methods and concepts.

**Conclusion:**

This study offers a unique insight into the barriers affecting cancer research and capacity-strengthening priorities from oncologists and researchers working in conflict-affected areas of the MENA region. These data will form the base for future capacity-strengthening initiatives addressing specific regional challenges.

## Background

The global cancer burden continues to rise in low- and middle-income countries (LMIC) (Table 1). By 2035, it is estimated that two-thirds of cancer cases will occur in developing countries, including countries in the Middle East and North Africa (MENA) region [1]. Multiple acute and chronic conflicts have affected the provision of cancer care services to populations in the MENA region and exacerbated existing challenges, including lack of universal health coverage, absent cancer control plans, inadequate human resource capacity and limited access to early detection and treatment options [2–7]. Furthermore, protracted armed conflicts across the region have directly led to mass migration and dismantlement of healthcare systems resulting in relative data poverty (Table 1) [13, 14].

Key mitigation strategies include multi-level interventions across the cancer care continuum, improving local health systems capacity, enhancing cancer registration and, importantly, generating contextually relevant data through research [15, 4]. Research plays an essential role in understanding disease burden, detecting geographical cancer patterns, assessing the success of interventions, addressing cancer health disparities and producing evidence-based health policies [16–18]. The relatively low regional research output in volume and quality has recently been highlighted [13]. Thus, it is imperative to mobilise individual, institutional, national and international efforts to strengthen cancer research capacity in conflict-affected MENA countries (Table 1). Against this backdrop, and to explore the factors affecting cancer research activity, we conducted key informant interviews (KIIs) aiming to understand the contextual landscape, barriers and enablers of cancer training, research and care. This work is part of the needs assessment phase of the Research for Health in Conflict in the Middle East and North Africa (R4HC-MENA) project efforts for capacity strengthening of cancer research in conflict settings within the MENA region [19].

## Methods

### Sampling and tools for data collection

A purposive sampling approach was used to identify key informants. We also employed a snowball sampling technique to help increase the number of respondents and used a semi-structured questionnaire to conduct face-to-face interviews with key informants. To establish a more holistic understanding of the impediments to cancer research, we interviewed key informants from various academic and clinical backgrounds, including oncology surgery, as well as individuals from non-governmental organizations (NGOs). Respondents originated from conflict-affected countries, including Lebanon, a refugee-host country, in addition to Syria and Iraq, both of which harbour active conflicts [20]. Participants were identified by the research team’s knowledge of cancer leaders in the three countries and the snowballing sampling method employed. At the end of the interviews, we asked the KIs to suggest key informant(s), who in their opinion could add valuable insights in line with the aims of the study. Interviews were done until data saturation was reached. Seventy-five informants were identified and sent invitations for interviews by e-mail, in English and Arabic, including a brief explanation of the rationale and aims of the study. Two reminder e-mails were sent to non-responders within two weeks of the first e-mail. Informants provided their consent through a form that was attached to the sent e-mail. Sixteen KIs responded to the email and were subsequently interviewed. The rest of the identified KIs did not respond to the emails we sent. At the start of each interview, verbal consent using the consent form for participating and recording the interview was obtained. Only one participant did not consent to recording the interview. On average, recorded interviews lasted 20 minutes.

After obtaining ethical approval from the American University of Beirut’s Institutional Review Board (IRB, Approval number: SBS-2018-0492) to conduct the interviews, a semi-structured guide was developed to gather contextual information on the clinical landscape, barriers and enablers of cancer training, research and patient care. Three researchers, ZAS, NEA and RAK, were involved in the interviewing process. ZAS and RAK developed the interview guide (supplemental document 1). The guide included six main questions that focused on (a) KIs’ perception of the individual and institutional barriers and enablers of cancer research and training and (b) the nature of training attended, given or recommended by the informants. The interview guide was developed in English and translated to Arabic by a certified translator and was piloted with 2 KIs and then revised. The interviews were conducted in English or Arabic, depending on the informant’s preference.

### Data analysis

Interviews were transcribed verbatim and then read several times by the researchers for familiarity with the collected data and for establishing a general thematic tone. Two researchers (ZAS and NEA) independently coded two interviews and described the coding book accordingly. All discrepancies in the coding were discussed between them, and the codebook was revised as needed. After agreeing on the coding structure, the rest of the interviews were coded by ZAS. The interview data were managed using the NVivo 12 software (QSR International), and thematic analysis was performed, as previously described [21]. Upon coding the transcripts segments into inclusive themes, we developed more specific secondary and tertiary sub-themes. The thematic analysis was then revised and finalised by ZAS and NEA. Direct quotations from the key informants are presented in italics to support and highlight key themes and findings.

## Results

**Figure 1.**
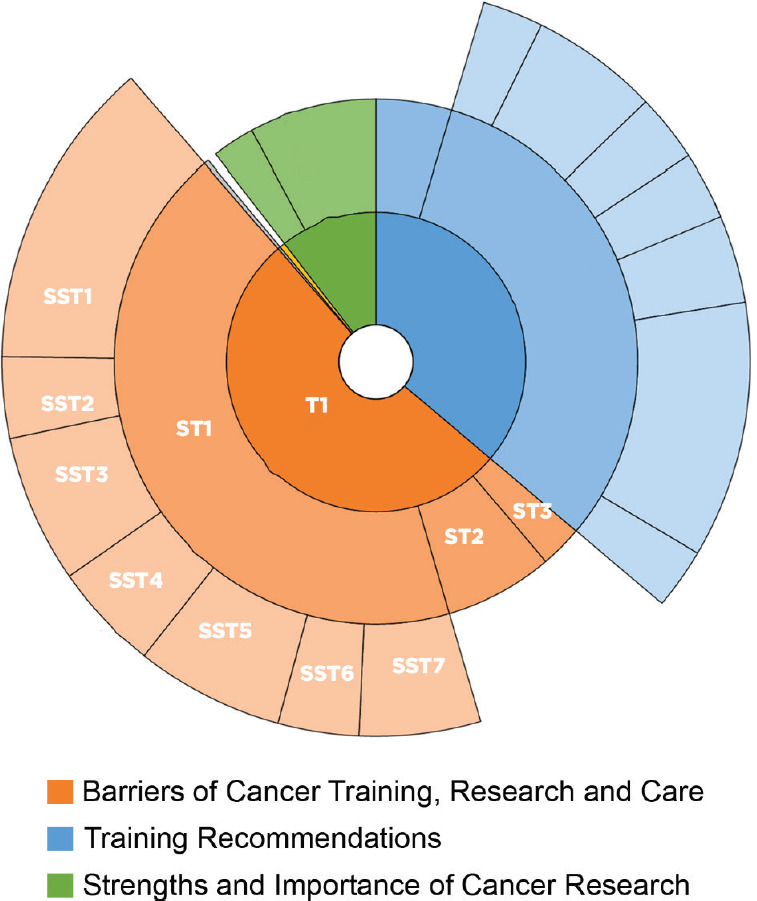
Hierarchical distribution of thematic analysis of Key Informant Interviews. Each colour represents a theme, which is dissected into sub-themes (ST) and sub-sub-themes (SST).

A total of 16 key informant interviews were conducted either in person (14 interviews), by telephone (1 interview) or using Skype (1 interview) between February and July 2019. Thematic analysis of the interviews yielded a rich description of the three major themes. First, key informants provided a detailed description of contextual barriers and challenges of cancer training, research and patient care. Interviewees also discussed the strengths and importance of cancer research. Finally, they gave recommendations regarding capacity strengthening activities, including cancer research training (Figure 1).

### Key informants demographics and training profile

**Figure 2.**
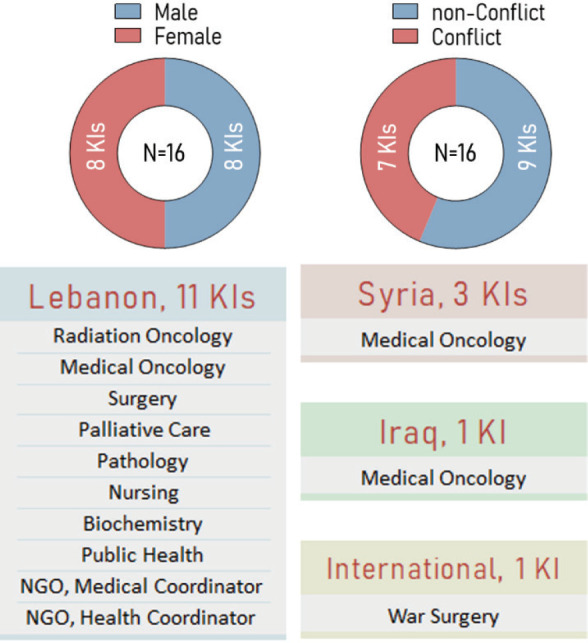
Key Informants’ profile, including gender, conflict status, country, and specialty distribution.

The KIs interviewed had extensive research portfolios in cancer prevention, clinical research, cancer education and basic cancer biology (Figure 2). The interviews also provided information about the capacity strengthening activities that were attended by the key informants. Five KIs mentioned that they attended the training including workshops or courses on topics like research ethics during their PhD, medical training or post-medical training. Twelve KIs delivered capacity strengthening activities touching on research methods, cancer control, capacity strengthening, advocacy, radiation oncology, clinical trials research ethics, clinical practise management and surgery. The nature of the delivered activities varied among KIs but included seminars, courses, workshops, face-to-face training and programs. Two KIs mentioned that offering capacity strengthening activities free of charge, describing their importance and stressing the need for such activities motivated people to attend. The breadth of the research and training profiles of KIs ensures the relevance and value of their perceptions and recommendations to the current study.

### Barriers to cancer care in the MENA (Theme 1)

Analysis of interviews revealed cultural, logistical and financial barriers to optimal cancer care in the MENA.

#### Cultural barriers

Few key informants focused on patients’ perception of cancer as a cultural barrier to cancer care and hence research, including paternalistic approach when disclosing cancer diagnosis and lack of understanding of the value of multi-disciplinary, informed cancer care.

**RID2**: ‘*Paternalistic approach*’ whereby ‘*the family or the head of the family must know before the patient’*.’

**RID4**: ‘*That people don’t understand the value of what we do even*’ when it comes to palliative care’.

**RID5**: ‘*Problems in the stigma, in understanding cancer in the population’.*’

#### Logistical barriers

In Syria and Iraq, cancer care barriers were heavily influenced by the active conflict plaguing both countries. This included the absence of equipment and medications, travel difficulties and security issues.

**RID11**: ‘*We used to have only one running machine, till the time of 2014 when ISIS invaded the city. Now, after liberation, that only machine is not working too*’ and ‘*many of the patients from….are either travelling to…or even to abroad to get treatment’.*’

**RID14**: ‘*Absence of ports…central lines…pumps*’ due to economic sanctions and patients travelling ‘*400-500 Km*’ to get to a cancer care centre.

**RID16**: ‘*Lack of drug. So, we give a patient a drug then we face a shortage of this drug. So, we would have to stop it,’* and ‘*in Syria, we rely on auto-transportation. Sometimes the road is dangerous, and there could be kidnapping, you know... Our centre was in a hot spot... So, the patient can pass away because of a bullet you know or rocket’*.*’*

#### Financial barriers

Few KIs detailed that financial difficulties prevent patients from accessing cancer treatment.

**RID3**: ‘*Most people either cannot afford the hospitalization or cannot afford the treatment, and that includes both Lebanese and non-Lebanese.*’

**RID6**: ‘*The clinical challenges are mainly related to two issues. One is the economy of the country and the amount or the number of patients who do not afford clinically’.*’

**RID7**: ‘*The main barriers to delivering cancer care are financial, and patients access to treatments…mainly the challenges patients mainly who don’t have any sort of financial coverage and have only coverage from the Ministry of Health because cancer treatments are so expensive’.’*

Interestingly, one KI mentioned that international NGOs operating in Lebanon, which provide limited support for cancer patients, face fundraising difficulties when it comes to cancer care since ‘*support for cancer patients might not be sustainable’,* given the high treatment cost and poor prognosis of some patients with cancer (**RID12**) [22, 3, 4].

#### Barriers of cancer research and training

Seven thematic barriers to cancer research were identified (Table 2). *(1) Funding and support*, which included a lack of financial resources and institutional support, were the most highly mentioned by interviewees. This was followed by *(2) human resource capacity*, which mainly focused on the lack of qualified personnel and insufficient research training. Importantly, KIs discussed the fragmentation of *(3) facilities* in Iraq and the lack of equipment and reagents in Syria. Furthermore, the *(4) lack of research culture*, including deprioritising cancer prevention, on the level of researchers, institutions and governments in countries of the MENA constituted the research culture barrier discussed by the KIs. Several key informants also reported difficulties in fostering *(5) collaboration* among institutions, organizations and countries. In addition to lack of collaboration, KIs highlighted multiple impediments that are related to *(6) conflict*, which included high patient drop-out rate due to the absence of drugs and personnel fleeing or affected directly by conflict-related violence. Lastly, international NGOs working for the benefit of the organization rather than the country was one of the *(7) political economy* barriers discussed. Example quotes of the aforementioned barriers are listed.

Barriers for conducting research training were similar to the research barriers and included insufficient time to conduct capacity strengthening activities, lack of funding and qualified personnel, and travel and safety issues due to conflict. Example quotes from two informants are found below.

**RID4**: ‘*Time is the biggest one and probably cost, but time is a big barrier, especially people in practice have limited time’. ‘We have challenges in the prevention in Lebanon because the government prefers to pay money for drugs and treating people and not putting his money in prevention’.*

**RID9**: ‘*Let’s say Gaza today, students, who say I have a scholarship waiting for me in Europe or the United States. I don’t have a visa…I can’t get out of Gaza and then get back in. Yemen, if you are in Aden, you could get out, where the coalition, the Saudi coalition. But if you are in Sanaa, how…do you get out. It is not obvious, and once you are out, how do you get back in’.*

### Strengths and importance of cancer research (Theme 2)

We extracted the perceptions of the strengths and importance of cancer research from the KIIs. Generally, KIs focused on the importance of research advancement in terms of incorporating research in the medical curricula, institutional support, availability of equipment and research capacity. Strikingly, all six informants who discussed research strengths were from well-known academic institutions in Lebanon. Examples highlighting the strengths of cancer research in the MENA include: ‘*At the institution, I think we deliver decent level and decent amount because we have the quantity and quality of research*’ **(RID5)** and ‘so I think Lebanon sort of does punch about its weight in terms of medical research in general and there is a lot of cancer research here’ **(RID 7)**. The importance of cancer research in improving clinical practice, lowering treatment costs, shaping policy and disseminating knowledge was highlighted by five informants. For example, KIs mentioned that producing data is important to ‘*get institutional support*’ **(RID4),** provides ‘*better understanding of our population may be of use of our population***’ (RID4)**, ‘*you prevent a disease it’s better than treating it. It’s less expensive, better outcome and better results*’ **(RID5)**; developing research stratified guidelines can help patients ‘to benefit from whatever they can get access to in the best cost-efficient way’ **(RID7)**; not publishing research is like ‘training for the marathon and not crossing the finish line’ **(RID1)**.

### Training Recommendations (Theme 3)

Informants were then asked to provide recommendations about the structure and nature of research training that would be ideal to develop, given the strengths and challenges prevalent in conflict settings. The results are summarised in Table 3. Briefly, most KIs found that an accredited face-to-face training on the postgraduate level on a variety of research topics is the optimal combination for research training. The online learning environment was not recommended by informants because of lack of internet access or lack of recognition of online courses in their country. KIs also preferred to give these training on the postgraduate level, specifically during the medical residency training. The topics that were recommended by informants included having workshops and sessions discussing qualitative and quantitative research methods, statistical analysis and conceptualising research projects. Selected quotes from informants discussing their topics preference are listed below.

**RID13:*** ‘I think a workshop that will tell these people how to start a good research project that will have an impact on the patient, it should be given, and that would entail a brief description of the importance of getting the appropriate clinical measures, the repository of biospecimen, the repository of diagnostic results including CTs and MRIs, and trying to do to force collaboration between people who are knowledgeable in all these’.*

**RID17***: ‘Epidemiology, data collection, ethical issues…it’s very lacking in lots of places. Some basic statistical analysis, critical literature, forming guidelines’.*

**RID9***: ‘The epidemiological is quantitative, but the functioning of a system for taking care of cancer patients is qualitative research, so you’re not involved in statistics and statistical analysis. You are involved in something else. You are involved in interviews and so forth and gathering other information. So, the epidemiological part is quantitative, but the system functioning is qualitative, and yes, people are given the basics’.*

Informants also provided valuable insight into how to motivate KIs to attend research training. They detailed that accrediting the training, providing practical training instead of didactic presentations and providing opportunities for networking, publishing, joining research projects, receiving mentorship and funding would be useful ways to motivate KIs to attend such training. Relevant quotes from informants are listed below.

**RID10**: ‘*You need to see the value of wise research importance. And I think one way that research is important to any healthcare professional is the fact that every guideline we have has come from some sort of research. So, making the courses appealing in terms of ‘understand your guidelines through research’ or ‘building upon the day to day work again tying it back to the research process’.’*

**RID17**: ‘*Probably, there had to be people interested, students, medical students, junior doctors have big motivation to publish and get involved in such projects. So, if you meet people by linking their mentors and projects, they can get involved’.*

**RID13**: ‘*Ask them to come up with their own questions so that they can discuss them in group and to see how we can implement these ideas in actual settings where you know we have the limited resources that you have and the limited budget that you have’.*

Lastly, informants were asked about ways of sustaining recommended research training. Eight KIs gave answers that focused on making such training an institutional priority, fostering collaborations among professionals, incentivising researchers, providing support from the government, securing funding and ensuring satisfactory outcomes. An example quote is listed below.

**RID2**:* ‘I think by creating collaboration among professionals, you would be able to sustain it. So, by having this link among different faculties from different universities would help to sustain this program of research, we can have a focus on oncology, and then we can have different sub-groups, and each group will be working on a topic, and then from there, you would generate more and more publications’.*

Other forms of research capacity strengthening activities were also recommended by many KIs. The informants argued that capacity strengthening should extend beyond training or courses into strengthening capacity for systems in place. For example, one informant discussed strengthening capacity for cancer centres on how to create a fully functioning system for cancer care, starting with receiving patients. Furthermore, strengthening capacity for cancer registration through completing the records and unifying data entry was mentioned by two KIs from Syria. Lastly, one KI highlighted that training should not only be limited to medical doctors but should also extend to strengthening capacity for nurses and paramedics in cancer and cancer research in conflict settings. Example quotes are listed below.

**RID10**: ‘*But, we really need to work on a research community in making the research more practical and more accessible. And that happens from an early design process of why are we even asking these questions, and why are we creating a document about research capacity, how is this really going to translate into something that’s going to better the community’s lives and better the individuals’ lives’.*

**RID13**: ‘*So, I understand when you are in the conflicting zone, you need to adapt to the environment, and one important thing is that you need to let alone all the major things that we can worry about and foster collaboration, so the first step is that we need to foster collaborations with existing institutions around the region so again we talk about Iraq and Syria, Lebanon will be a place, Jordan will be a place where they can help with, and the starting point you know is to train personnel using the system built-in these institutions in Jordan and Lebanon and try to align with their goals and standards so that at least you start building a system that will work there’.*

## Discussion

The underlying contextual landscape and barriers to health research in general, let alone cancer research, training and clinical care in conflict-affected MENA countries are not well understood [23, 24]. To this end, we sought to explore these barriers by conducting interviews with key informants working in conflict-affected environments in the region. The themes emerged from these interviews are in accordance with those listed in the conceptual framework designed by colleagues at R4HC-MENA for the capacity strengthening of health research in conflict-affected MENA countries (Table 2, [23]). However, even within these themes, there are specificities of cancer research, training and clinical care in such settings which are not captured in the framework and thus need to be highlighted.

For instance, KIs from Iraq and Syria examined the detrimental impact of conflict on the delivery of cancer care. This included inaccessibility of cancer care facilities due to blocked and unsafe roads, equipment and drug shortages due to economic sanctions and scarcity of cancer care centres. The impact of conflict on the provision of healthcare in Iraq is not new. The Iraqi health system was considered the best in the Middle East before 1980, but recurrent conflicts and economic sanctions, starting with the war against Iran to the civil war in 2014, have dismantled the health sector, including cancer care infrastructure and human capacity [25, 26]. In Syria, the recent civil war has caused a progressive increase in poverty and a decrease in healthcare provision, including cancer care. For example, essential cancer diagnostic and therapeutic tools like tomography scanners and radiation therapy only exist in Damascus and Latakia, which restricts access to millions of people under siege or in remote areas. When accessible, some services require out-of-pocket payment, which may be inaccessible to many given the loss of wealth endured by the Syrian population. These barriers result in a delay in early detection, diagnosis and treatment. Indeed, many reports document that most cancers emanating from many MENA countries are diagnosed at later stages and affect a younger population when compared to the world average [8–12]. This highlights the importance of contextualised strengthening of the capacity for cancer prevention in the MENA. Next, informants highlighted impediments for cancer research and training, which ranged from inadequate support and funding to weak human resource capacity, fragmented facilities, absence of research culture and weak collaborative efforts. Importantly, conflict-related barriers were also explored by KIs, and they included the sudden absence of drugs during research, lack of quality control over drugs and fleeing of personnel. These interviews highlight the unique nature of barriers experienced by oncologists and researchers in conflict settings to conduct research, attend training and deliver cancer care.

Strengthening capacity for cancer research through training workshops or courses should account for these contextual challenges. To this end, we asked informants to recommend the nature of capacity strengthening activities given the context of conflict. They recommended that an accredited face-to-face training on the postgraduate level on topics, including research methods, conceptualising research projects and statistical analysis, is the optimal combination for research training. Lastly, such activities should take into consideration existing capacity, which was also mentioned by informants from Lebanon, where research capacity is strong [13]. Previous studies aimed at understanding the need for courses to enhance research skills have been conducted in conflict-affected settings. Specifically, researchers at Birzeit University in Palestine have established the need for a capacity building course focused on mental and psychosocial health research [27]. Our study and the study by Birzeit University are an integral part of the contextualization of the assessment, success and sustainability of capacity strengthening in such settings. Indeed, setting research agendas in LMICs by international funders hinders capacity strengthening and promotes research that aligns with funders’ agenda rather than countries’ capacity needs [28–31]. This was also highlighted as a political economy barrier for cancer research by one of the key informants (Table 2).

It is crucial to consider the limitations of the study conducted. First, the results are limited to the key informants who agreed to be a part of the study and the sample may not be representative of all viewpoints. Many insights regarding the barriers and recommendations for cancer care and research might have been missed as a result. For example, it would have been important to interview employees in the public sector, especially at the Ministry of Public Health to highlight the barriers from the perspective of the health system as well. Second, KIs came from different academic and clinical backgrounds, and while this provides a more inclusive understanding of the aims of the study, it might weaken the study findings since their perception of the needs might be different and potentially conflicting.

## Conclusions

Many studies aimed at understanding the needs for cancer care and research have been conducted within the MENA context and beyond [32, 18]. However, to our knowledge, this is the first study to explore, in depth, the needs and recommendations of oncologists and cancer researchers in conflict-affected settings. The study is a part of the need assessment phase of the cancer workstream within the R4HC-MENA project that aims to strengthen research capacity in conflict-affected areas. Importantly, this work is driven by countries within the conflict-affected MENA region, which is essential for ensuring the success of the capacity strengthening phase. It was found that barriers for cancer training, care provision and research in conflict-affected MENA countries are multi-faceted. Institutional, national and international efforts are sorely needed to alleviate the challenges facing cancer research and training in conflict settings in the MENA region. This work will serve as a primer for future R4HC project needs assessment initiatives focusing on identifying the training needs for cancer research in conflict-affected MENA countries. Upon finalising the needs assessment phase, we aim to launch evidence-based capacity strengthening activities for cancer research in the MENA region.

## Author contributions

Substantial contributions to the conception or design of the work and/or the acquisition, analysis or interpretation of data for this work were done by ZAS and DM. Drafting the work or revising it critically for important intellectual content was done by all authors. Final approval of the version to be published was provided by all authors.

## Conflicts of interest

The authors have no conflicts of interest to declare.

## Ethics and consent

Ethical approval was obtained from the Institutional Review Board at the American University of Beirut (IRB approval number: SBS-2018-0492). All participants consented for being interviewed. Participants who were recorded gave their consent to being recorded.

## Funding information

This publication is funded through the UK Research and Innovation GCRF Research for Health in Conflict in the Middle East and North Africa (R4HC-MENA) Project, developing capability, partnerships and research in the Middle and North Africa ES/P010962/1.

## Figures and Tables

**Table 1.** Key definitions included in the manuscript text.

Key definitions
**LMIC (Low- and Middle-Income Country):** According to the World Bank’s definitions, drawing on 2020 figures, low-income economies have a gross national income (GNI) per capita of $1025 or less; the GNI per capita of lower middle-income is between $1025 and $3995; and upper middle-income economies have a GNI per capita of between $3996 and $12,375 [33].
**Capacity strengthening:** A process of developing, upgrading and/or expanding pre-existing capabilities at the individual, organisational and institutional levels to plan, conduct and disseminate evidence-based knowledge[34].
**Conflict and conflict-affected:** Conflict, as used here, refers to a violent armed struggle between hostile groups, resulting in over 25 battle-related deaths per year [35]. We use conflict-affected in accordance to the World Bank’s definition to indicate areas that may not be bearing the brunt of violence, as it is the case in Lebanon, but still experience high levels of institutional and social fragility, as a result of conflict, e.g., in the form of an influx of refugees or internally displaced populations[36].
**Protracted conflict:** Hostile interactions which extend over long periods of time with sporadic outbreaks of open warfare fluctuating in frequency and intensity [37].
**MENA (the Middle East and North Africa) Region:** Covers 24 countries, namely, the 21 members of the Arab League (Algeria, Bahrain, Djibouti, Egypt, Iraq, Jordan, Kuwait, Lebanon, Libya, Mauritania, Morocco, Oman, Palestine, Qatar, Saudi Arabia, Somalia, Sudan, Syria, Tunisia, the United Arab Emirates and Yemen), as well as Iran, Israel and Turkey [38].

**Table 2.** Thematic barriers of cancer research.

Thematic Barrier	Frequency	Example quotes
Support and funding	13 KIs	**RID2:** *I think it all goes back to funding, if you can find funding to buy your time, then you can do research. You cannot find funding to buy your time; then, this is an issue with all the hassles of being a faculty.* **RID16:** *When you have your paper done, you’re going to submit for international journals, and the fee is very high! (1500 $ at least). So, if you calculate to Syrian pounds, it’s a lot of money. So, the problem is also the cost of publishing a paper. That’s why I published in our university journal because it’s free. That’s why if you google it you can’t find it in some local paper*
Human resources capacity	9 KIs	**RID11:** *One of the difficulties is the absence of qualified clinician-scientists, and this might be due to many reasons.* **RID13:** *You can have the skilful person you want to have, but if you don’t have the appropriate training, you will end up losing everything, so the best way to have a system working is to really have the infrastructure plus the training of the skilful persons to come up with a project that will materialize sooner or later*
Facilities	6 KIs	**RID11***: What I want to achieve is a real comprehensive cancer care system in Iraq. What we have is mostly fragmented facilities here and there* **RID14**: *we need PCR with sequential we need flu psychometry, we need many things for research, for lab* **RID16**: *In our centres, for example, we have over 500 beds. In our breast service, a month, over 3000 patients present to our section to receive treatment. Only to the breast service*
Research culture	6 KIs	**RID4:** *…we are not strategic in the way we choose to act … in a crisis in what we do to jump to deliver a service instead of setting up a research to get evidence to see what we do… We have challenges in the prevention in Lebanon, because the government prefers to pay money for drugs and treating people and not putting his money in prevention* **RID14:*** The second point into the barriers that are there is that we still have a taboo in the society that we don’t talk about cancer, and I guess this is major, even though people are aware and the knowledge is high, this kind of taboo is really preventing people, even physicians and researchers, to come up with new regimens to understand better the disease, to come up with let’s say new research ideas, and I guess this is something that should be tackled at the level of the institution, at the level of the government, at the level of each individual involved in that.* **RID6:*** The second problem we have is the interest in research. We have at least from the medical side, not all physicians or medical teams are fully interested in medical research, and they are more focused on clinical work.*
Collaboration	6 KIs	**RID10:** *So I think one of the biggest barriers on a country level is buying from the healthcare NGO-s that are supposed to be working together but yet the reality is that they’re working individually.* **RID16:** *I think collaboration could help with funding!*
Conflict	5 KIs	**RID16:** *These patients are enrolled in our research! Research, we study cancer drugs sometimes. Suddenly, we don’t have the drug anymore. The patients enrolled in the research (clinical research) cannot receive their treatment! A third one is the source of our drug. It’s not the original one. From eastern countries. We don’t know if it has the same quality.* **RID9:*** Salaries don’t get paid, supplies do not get distributed, equipment is not well prepared, personnel flee or killed or injured, and therefore especially in lower middle-income countries where you may have already a lack of sufficient medical personnel in peacetime the number even decreases.*
Political economy	4 KIs	**RID10:** *And one of the barriers that I’ve seen with research specifically between NGOs and UN-related groups as well is how much ownership over data people have. So in a community, especially in Lebanon, in a community that values the privacy of data and values an organization’s individual goals over the country’s goals or the people’s goals, it’s very hard to get buy-in from these stakeholders to build research capacity unless there is a clear benefit for them individually before the benefit of the whole.* **RID1:*** We can be part of clinical trials that involve multiple centres, yes we can be part of the global you know research landscape, but as a moving force, we are not on the map…*

**Table 3.** Training recommendations by informants.

Training criterion	Recommendation	Frequency	Example quote
Learning environment	Face to Face (e.g., workshop)	5 KIs	**RID1**: ‘*workshops where small groups that are mentored by experts and experts provide experience. I think personal human interaction is much more conducive to a productive learning experience.’*
	Online	1 KI	**RID7**: ‘*Probably more of an online environment. If we are targeting busy positions and students that can be participating because they are interested.’*
	Blended (online + Face to Face)	2 KIs	**RID11**: ‘*The blended one because the people here they are not well believing in online learning, even the University of Higher Education in Iraq is still not recognising degrees in online courses. Even when I returned back, I did not submit the Master of Science in Advanced Oncology from Ulm University in Germany to be qualified in Iraqi universities.’*
Career stage	Undergraduate	2	**RID2: ‘***I think in order to have a culture of research, this idea has to be stilled in student since undergraduate, so we have to start in undergraduate program start to train students.’*
	Postgraduate	8	**RID3: ‘***I think they need post-graduate training on research.’*
	All levels	1	**RID11:** *I think to all levels because we need to establish the culture of research, which is not that well-focused even in medical education. So, we need to push the medical research at all levels, starting from the undergraduate medical education through postgraduate education through continuous medical and professional education and development. So, in all aspects, we need to push toward this culture of medical research. We need to establish people who are clinician-scientists, who are devoting like half of their time into research and the other half into clinical practice, which is now not available, unfortunately.*
Accreditation	Yes	5	**RID11:** *Yes, because when the courses are accredited, the clinicians or the medical students will be encouraged to attend, while if it is not accredited, they will not be regarded as important as the accredited.*
	No	2	**RID10:** *I think we need to be very careful with accreditation because sometimes accreditation can limit a speciality versus enable it, especially if we’re trying to build research capacity on a national level. Putting in an accreditation value will limit how many people will participate in it, even though we can get more people to be part of the research process through making it so wanted.*
